# Reduced social brain coupling to others’ face threats in narcissism

**DOI:** 10.1093/cercor/bhag175

**Published:** 2026-10-27

**Authors:** Matias Piispanen, Jarno Tuominen, Mariel Wuolio, Pentti Henttonen, Ville J Harjunen, Emmi Koskinen, Elizabeth Krusemark, Enrico Glerean, Pärttyli Rinne, Mikko Sams, Anssi Peräkylä, Juha Salmi

**Affiliations:** Department of Neuroscience and Biomedical Engineering, Aalto University, Rakentajanaukio 2 C, 02150 Espoo, Finland; Faculty of Social Sciences, University of Helsinki, Unioninkatu 37, 00170 Helsinki, Finland; Aalto NeuroImaging, Aalto University, Otakaari 5 I, 02150 Espoo, Finland; MAGICS–Aalto, Aalto University, Otakaari 2 A, 02150 Espoo, Finland; Department of Neuroscience and Biomedical Engineering, Aalto University, Rakentajanaukio 2 C, 02150 Espoo, Finland; Faculty of Social Sciences, University of Helsinki, Unioninkatu 37, 00170 Helsinki, Finland; Department of Psychology and Speech-Language Pathology, University of Turku, Assistentinkatu 7, 20014 Turku, Finland; Faculty of Social Sciences, University of Helsinki, Unioninkatu 37, 00170 Helsinki, Finland; Helsinki Institute for Social Sciences and Humanities, University of Helsinki, Fabianinkatu 24 A, 00014 Helsinki, Finland; Helsinki Institute for Social Sciences and Humanities, University of Helsinki, Fabianinkatu 24 A, 00014 Helsinki, Finland; Faculty of Social Sciences, Tampere University, Kalevantie 5, 33100 Tampere; Finland; Faculty of Medicine, University of Helsinki, Haartmaninkatu 8, 00290 Helsinki, Finland; Faculty of Social Sciences, University of Helsinki, Unioninkatu 37, 00170 Helsinki, Finland; Psychology and Neuroscience Department, Millsaps College, 1701 North State Street, Jackson, MS 39210, United States; Department of Neuroscience and Biomedical Engineering, Aalto University, Rakentajanaukio 2 C, 02150 Espoo, Finland; Department of Neuroscience and Biomedical Engineering, Aalto University, Rakentajanaukio 2 C, 02150 Espoo, Finland; Department of Neuroscience and Biomedical Engineering, Aalto University, Rakentajanaukio 2 C, 02150 Espoo, Finland; MAGICS–Aalto, Aalto University, Otakaari 2 A, 02150 Espoo, Finland; Faculty of Social Sciences, University of Helsinki, Unioninkatu 37, 00170 Helsinki, Finland; Department of Neuroscience and Biomedical Engineering, Aalto University, Rakentajanaukio 2 C, 02150 Espoo, Finland; Unit of Psychology, Faculty of Education and Psychology, University of Oulu, Erkki Koiso-Kanttilan katu 1, 90570 Oulu, Finland

**Keywords:** empathy, functional magnetic resonance imaging, intersubject correlation, narcissism, naturalistic neuroscience

## Abstract

Although several studies have linked narcissism to reduced empathy, the neural mechanisms underlying narcissistic behavior in socially complex, naturalistic contexts remain poorly understood. This study investigated how grandiose narcissistic traits are associated with neural dynamics in empathy-related areas of the “social brain” when participants observed another person receiving face-threatening feedback. We hypothesized that individuals with high narcissistic traits would exhibit lower intersubject correlation (ISC), indicating less synchronized neural responses across participants, in the anterior insula (aINS), dorsolateral prefrontal cortex (dlPFC), and posterior cingulate cortex (PCC) in response to face threats directed at others. Participants with high (*n* = 28) and low (*n* = 30) scores in grandiose narcissism watched videos during functional magnetic resonance imaging in which another person received face-threatening feedback to a personal story. Consistent with our hypothesis, highly narcissistic individuals exhibited reduced ISC in the dlPFC and PCC. In aINS, the effect did not reach statistical significance. The present findings suggest that trait narcissism reduces convergence in empathic responses particularly in areas related to cognitive empathy when witnessing social threats targeted at others. These findings further indicate that ecologically relevant experimental designs may help clarify how personality traits are expressed in neural responses to socially meaningful events.

## Introduction

Narcissism—whether viewed as a continuum of traits in the general population or in its pathological form—is understood as disproportional preoccupation with self. While the person’s relation to him/herself is thus at the core of narcissism, it also shapes how individuals relate to others (Morf and Rhodewalt 2001). The interpersonal strategies to enhance self-image in narcissism operate at the expense of others (Grapsas et al. 2020). Reflecting a self-serving interpersonal style, in the current version of the Diagnostic and Statistical Manual of Mental Disorders (5th ed.; DSM-V; American Psychiatric Association 2013), one of the key characteristics of Narcissistic Personalit Disorder is a lack of empathy, described as being “unwilling to recognize or identify with the feelings and needs of others” (American Psychiatric Association 2013: 670).

Narcissism can take the form of grandiosity, involving feelings of superiority, and vulnerability, involving an insecure need for approval of the self (e.g. Sedikides 2020). While narcissism’s problematic self-focus and self-serving interpersonal strategies are well established (Morf and Rhodewalt 2001; Grapsas et al. 2020; Eddy 2021), its neural underpinnings remain poorly understood. This concerns particularly social interaction, where narcissistic features are most prominently observed. Examining individual differences in neural dynamics when people observe complex social interactions may help clarify how narcissistic traits are associated with differences in the moment-to-moment processing of socially relevant events.

Early clinical observations suggested that narcissistic individuals tend to perceive others primarily in terms of how they serve their own self-related needs (Kernberg 1975; Kernberg 1991). Contemporary research on empathy further supports this view. Meta-analyses of behavioral data suggest that grandiose narcissism is associated with lower affective empathy (the ability to feel another person’s emotions) measured via self-reports or experimental tasks (Urbonaviciute and Hepper 2020; Simard et al. 2023). In cognitive empathy (the capacity to understand another person’s thoughts and feelings) the picture is different: persons with high grandiose narcissism do self-report poor cognitive empathy, but in cognitive empathy tasks, they actually perform equally to those with low narcissism cores (Urbonaviciute and Hepper 2020). This suggests that while narcissistic individuals with grandiose self-concept may possess the capacity for cognitive empathy, they lack the motivation to use it. Narcissistic individuals often prioritize self-enhancement over affiliation, using social interactions primarily to reinforce their self-image and seek external validation, rather than to build supportive or genuine connection (Morf and Rhodewalt 2001; Grijalva and Zhang 2015; Mahadevan et al. 2019; Chen et al. 2024; Mahadevan 2024). Given that narcissistic individuals are proposed to use social interactions as a key arena for self-promotion (Morf and Rhodewalt 2001), it is of particular importance to examine narcissism in interactive settings.

Recently, the interactional sociology of Erving Goffman (1959) has been taken up in the study of narcissism in social interaction (Peräkylä 2015). In Goffman’s view, a person in interaction indirectly claims a self with attributes: by acting with others, we also present ourselves as a particular kind of person, for example, as competent, confident, trustworthy, or knowledgeable about the things that we talk about. This claimed self is vulnerable, as it may become discredited when others do not validate it. In a string of Goffman-inspired studies, Koskinen et al. (2024a, 2024b, 2025), along with related work by Harjunen et al. (2023), have shown that especially persons with grandiose narcissism respond physiologically and behaviorally in elevated ways to interactional situations where their projected self is put under threat. However, interactional threat is not limited to one’s own self-presentation. Goffman (1956) suggested that the discreditation of self in interaction affects emotionally not only the discredited person themself, but also those who witness the moment of discreditation: those present experientially share the embarrassment. No studies have yet examined how narcissistic individuals with grandiose self-concept respond to others’ face-threatening experiences and how this is mirrored in their empathy-related neural activity.

Prior neuroscientific research indicates that narcissistic individuals are neurally responsive to their own social pain, yet less engaged when processing others’ distress. For instance, in a simple social exclusion paradigm, individuals high in grandiose narcissistic traits exhibited heightened activation in the anterior insula (aINS) and dorsal anterior cingulate cortex when excluded (Cascio et al. 2015). In contrast, other studies report reduced activation in these same areas during affective empathy tasks (e.g. Jauk and Kanske 2021). For instance, an fMRI study by Fan et al. (2011) found that individuals with high levels of narcissism showed weaker activity in the right aINS during a task where they were asked to empathize with emotional pictures of faces. Similarly, a study by Scalabrini et al. (2017), focusing especially on grandiose narcissism, showed that narcissistic traits are associated with reduced aINS activation when participants anticipate human touch directed at themselves. However, it remains unclear whether brain responses to viewing static images under explicit instructions to empathize, or to anticipating touch, both outside real-world social contexts, truly capture the essential aspects of neural processes that give rise to narcissistic behaviors in real-world interactive settings.

Addressing this limitation, Jauk et al. (2024) conducted a pioneering study where they recorded brain activation in individuals with higher vs. lower narcissism scores when instructed to empathize with other people telling short autobiographical stories about situations when they had experienced loss or threat. They found that, in individuals with high grandiose narcissistic scores, the subjective experience of compassion in response to the story was associated with lower activation in the right ventral striatum and left aINS, whereas no corresponding differences were observed in social cognition. However, in everyday encounters, individuals are often not only recipients or targets of others’ experiences but also witnesses to how those experiences are responded to by third parties. Such triadic situations are particularly consequential when they involve the public evaluation of another person, as they can give rise to shared embarrassment and vicarious social threat (Goffman 1956). Importantly, these socially evaluative contexts are especially relevant for individuals high in grandiose narcissistic traits, whose sensitivity to social status and evaluation is well established (e.g. Somerville et al. 2010; Eisenberger et al. 2011; Thomaes et al. 2011; Heitmann et al. 2014; Harjunen et al. 2023).

The present study examined empathy-related neural responses in narcissistic individuals while they witnessed a third party threaten another person’s self-presentation. The research participants witnessed another individual receiving feedback on a personal story while being scanned with fMRI, without explicit instructions to empathize, in a context more closely resembling real-world interactive settings. We introduce a novel approach to narcissism research by employing intersubject correlation (ISC; Hasson et al. 2004), a method for assessing the similarity of regional brain activity time courses across individuals presented with the same stimulus. ISC is particularly well suited for studying complex and dynamic naturalistic stimuli, as it captures shared stimulus-driven neural responses without requiring predefined event structures or assumptions about the underlying cognitive processes. Previous studies have shown the utility of ISC in characterizing individual differences in social and affective processing. For example, individuals with autism spectrum disorder show reduced ISC in brain regions related to social cognition, such as the aINS and the posterior cingulate cortex (PCC), when viewing naturalistic social stimuli (Salmi et al. 2013). Traditional task-based fMRI approaches typically rely on the general linear model (GLM) and use brief, minimalistic, and isolated tasks, capturing inadequately the complexity of real-world interactions. Moreover, unlike GLM-based approaches that rely on predefined stimulus models, ISC captures shared stimulus-evoked neural dynamics without requiring explicit assumptions about the timing or nature of the underlying cognitive processes, making it especially suitable for complex naturalistic stimuli. While previous ISC studies have often employed third-person paradigms in which participants observe movies or narratives (Hasson et al. 2010; Jääskeläinen et al. 2021), the present paradigm simulates an unfolding interpersonal situation in which social meaning emerges through the interaction between the narrator and the evaluator. The paradigm captures a relational event, placing it conceptually between traditional third-person social cognition paradigms and the interaction-focused framework of second-person neuroscience (Schilbach et al. 2013; Redcay and Schilbach 2019).

While the broader pre-registered experiment included multiple research aims, the present paper focuses on a preregistered hypothesis (H2) concerning empathy-related neural responses. Building on previous findings that individuals high in narcissistic traits show reduced activation in the aINS, PCC, and dorsolateral prefrontal cortex (dlPFC) during empathy tasks (Fan et al. 2011; Scalabrini et al. 2017; Jauk et al. 2024), we sought to examine whether trait narcissism is linked to how people react to others’ face threat and how grandiose traits uniquely modulate neural activation associated with affective empathy and perspective-taking. Specifically, we predicted that individuals with high levels of grandiose narcissistic traits (N+), compared to those with low levels (N–), would exhibit reduced ISC and lower activation in the aINS and dlPFC (H2.1), as well as in the PCC (H2.2), while viewing others’ face-threatening feedback (see osf.io/bfj3u). We focused on grandiose narcissism because neuroscience research has predominantly examined this dimension (Jauk and Kanske 2021), and because the physiological evidence is more consistent for grandiose than vulnerable narcissism in social-evaluative contexts (Lobbestael et al. 2014; Jauk et al. 2024; Koskinen et al. 2024a).

## Materials and methods

### Participants

Participants were recruited via university mailing lists, social media and flyers and selected through a prescreening procedure. Inclusion criteria were age ≥18, right-handedness, no psychiatric or neurological diagnoses, and no MRI constraints. Altogether 935 participants completed the online prescreening questionnaire. Those who scored in the higher end (> 40) of the Narcissistic Personality Inventory (NPI–13; Gentile et al. 2013) scale for trait narcissism were approached with scheduling (group N+), together with a corresponding control group on the low range scores (<27) (group N–). The cut-off scores were selected based on previous literature (Henttonen et al. 2022), and in this sample selected participants either above the 80th percentile or below the 30th percentile range. In addition, we included the first participant who completed the experiment in the N– group. This individual scored slightly above the pre-set cut-off (NPI-13 = 29). At the time of the data collection, there were no studies using the ISC method in this population to estimate the effect size, and thus instead of power analyses we targeted a conventional sample size of an fMRI study with approximately 35 participants per group. The N+ group included 36 individuals (25 females, 11 male, mean *±* SD age 29.2 *±* 8.87 years, age range 20–49 years, 43.2% had a BA or higher degree) and the N– group included 37 participants (25 females, 12 males, mean *±* SD age 27.4 *±* 6.83 years, age range 19–44 years, 47.2% had a BA or higher degree). The final sample comprised 28 individuals in the N+ group and 30 individuals in the N– group, after excluding participants due to excessive head motion (see fMRI preprocessing section). All participants gave written informed consent, and the study was approved by the ethical board of Aalto University (D/1223/03.04/2022). The study was preregistered (osf.io/bfj3u).

### Psychological measures

In the pre-screening survey participants answered several questionnaires. In this study, we included gender, age, education, and scales on grandiose narcissism and empathy. The NPI-13 with a 5-item Likert scale (1 = Totally disagree, 5 = Totally agree) was used to assess grandiose narcissism. Cronbach’s alpha for the NPI-13 in this sample was 0.944. The Brief Interpersonal Reactivity Index (bIRI; Ingoglia et al. 2016), consisting of four subscales, measuring personal distress (bIRI-PD), perspective taking (bIRI-PT), empathic concern (bIRI-EC), and fantasy (bIRI-F), was used to evaluate empathy. Cronbach’s alpha for the bIRI subscales in this sample were 0.731 for empathic concern, 0.714 for personal distress, 0.771 for perspective taking, and 0.790 for fantasy.

### Experimental procedure

The experiments were conducted at the ABL and AMI Centre, Aalto NeuroImaging, Aalto University School of Science. The following describes the data collection procedure used in this experiment. For an extended procedure including an additional experiment carried out during the imaging session, see osf.io/bfj3u. During fMRI scanning participants watched videos of another person (in reality, an actor) telling four personally meaningful stories of either being admired or being excluded, both recently and in their youth, followed by videos of either face-affirming (i.e. supportive the tellers self-presentation; Goffman 1955) or face-threatening (i.e. challenging their self-presentation) feedback (see Fig. 1). Participants were not given explicit instructions to empathize or to refrain from empathizing and they were simply instructed: “Watch the feedback received by another person.” The present study focuses on neural responses to the face-threatening feedback, while ISC analysis results for the admiration feedback are reported in Supplementary material. After each feedback video, participants answered four Self-Assessment Manikin (SAM; Bradley and Lang 1994) questions on four dimensions (emotional valence, arousal, control, pride) rated 1–9, as well as the position of their locus of attention (1: provider of feedback, 9: teller or own experience) on a visual-analogue scale assessing the other–self dimension (was your focus primarily on the self or the other). See Supplementary Material 1 for a detailed description of stimulus preparation, Supplementary Material 2 for the feedback transcriptions and Supplementary Material 3 for the SAM scales.

**Figure 1:**
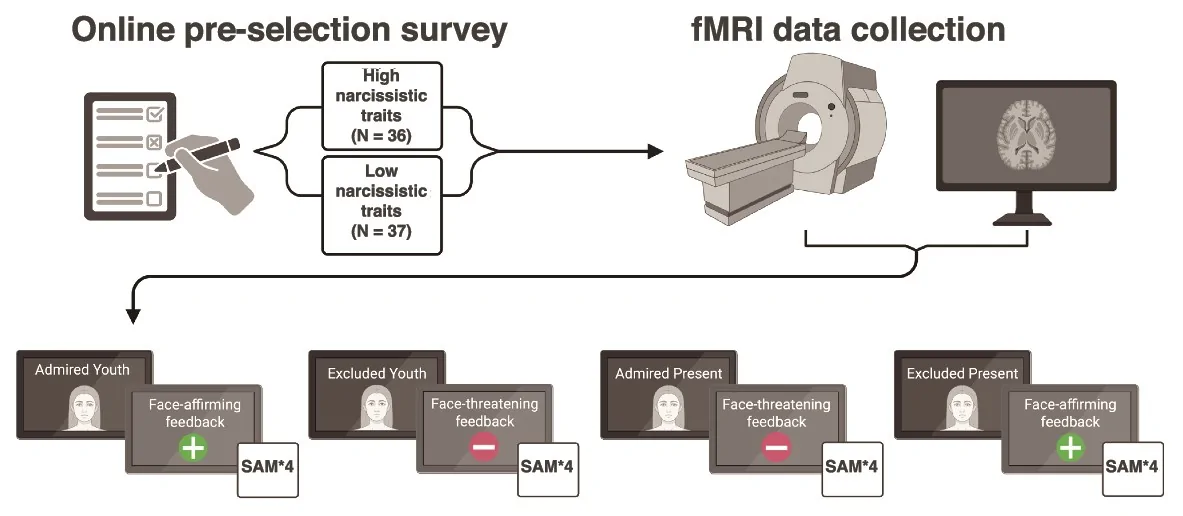
Outline of the study procedure. Participants were selected based on an online survey for fMRI imaging. In the experiment, they viewed four narrative videos depicting situations in which the narrator had felt either admired or excluded, one from their youth and one from the present. The video was followed by either face-affirming or face-threatening feedback, and a rating of self-state using self-assessment manikin (SAM). Our main analyses focus on the face-threatening feedback, with face-affirming feedback reported in the Supplementary material. Additional data not used in this study is not portrayed. Created with biorender.com.

### fMRI data acquisition

Functional and structural MRI data were acquired on a Siemens MAGNETOM Skyra 3 T scanner equipped with a 30-channel head coil. Each scanning session began with a localizer, followed by the first run, which included videos of others’ narratives, feedback videos, and the SAM questionnaires. A 6-min anatomical scan was acquired at the end of the session. The whole-brain blood oxygenation level-dependent (BOLD) functional data were acquired using a simultaneous multi-slice echo-planar imaging sequence with fat suppression, a multiband acceleration factor of 4, repetition time (TR) = 594 ms, echo time (TE) = 16 ms, a flip angle of 50°, a slice thickness of 3 mm, an in-plane resolution of 3 × 3 mm, 44 axial slices, a 64 × 64 matrix, anterior–posterior phase encoding and a field of view (FOV) of 192 mm. The structural images were collected using a T1-weighted magnetization-prepared rapid gradient-echo sequence with TR = 2530 ms, TE = 3.3 ms, a flip angle of 7°, a slice thickness of 1 mm, an in-plane resolution of 1 × 1 mm, a distance factor of 50%, 176 sagittal slices, a 256 × 256 matrix, anterior–posterior phase encoding, an FOV of 256 mm, and an acquisition time of 6 min 2 s. During fMRI scanning, participants’ pulse and breathing were recorded.

### fMRI preprocessing

Results included in this manuscript come from preprocessing performed using FMRIPREP version 20.2.0 (Esteban et al. 2018, RRID:SCR_016216), a Nipype (Gorgolewski et al. 2011; Gorgolewski et al. 2017, RRID:SCR_002502) based tool. Each T1w (T1-weighted) volume was corrected for INU (intensity non-uniformity) using N4BiasFieldCorrection v2.1.0 (Tustison et al. 2010) and skull-stripped using antsBrainExtraction.sh v2.1.0 (using the OASIS template). Brain surfaces were reconstructed using recon-all function from FreeSurfer v6.0.1 (Dale et al. 1999, RRID:SCR_001847), and the brain mask estimated previously was refined with a custom variation of the method to reconcile ANTs-derived and FreeSurfer-derived segmentations of the cortical gray-matter (GM) of Mindboggle (Klein et al. 2017, RRID:SCR_002438). Spatial normalization to the ICBM 152 Nonlinear Asymmetrical template version 2009c (Fonov et al. 2009, RRID:SCR_008796) was performed through nonlinear registration with the antsRegistration tool of ANTs v2.1.0 (Avants et al. 2008, RRID:SCR_004757), using brain-extracted versions of both T1w volume and template. Brain tissue segmentation of cerebrospinal fluid (CSF), white-matter (WM) and GM was performed on the brain-extracted T1w using fast (Zhang et al. 2001) (FSL v5.0.9, RRID:SCR_002823).

Functional data was slice-time corrected using 3dTshift from AFNI v16.2.07 (Cox 1996, RRID:SCR_005927) and motion corrected using mcflirt (FSL v5.0.9; Jenkinson et al. 2002). This was followed by co-registration to the corresponding T1w using boundary-based registration (Greve and Fischl 2009) with six degrees of freedom, using bbregister (FreeSurfer v6.0.1). Motion correcting transformations, BOLD-to-T1w transformation and T1w-to-template warp were concatenated and applied in a single step using antsApplyTransforms (ANTs v2.1.0) using Lanczos interpolation.

Physiological noise regressors were extracted using CompCor (Behzadi et al. 2007). Principal components were estimated for the two CompCor variants: temporal (tCompCor) and anatomical (aCompCor). A mask to exclude signals with cortical origin was obtained by eroding the brain mask, ensuring it only contained subcortical structures. Six tCompCor components were then calculated including only the top 5% variable voxels within that subcortical mask. For aCompCor, six components were calculated within the intersection of the subcortical mask and the union of CSF and WM masks calculated in T1w space, after their projection to the native space of each functional run. Frame-wise displacement (Power et al. 2014) was calculated for each functional run using the implementation of Nipype.

Eight participants from the N+ group and seven participants from the N– group were removed due to excessive head motion (i.e. more than 0.5% of the volumes of interest showed frame-wise displacement exceeding 0.5 mm). The excluded participants did not differ from the included in age or narcissistic traits (see Supplementary Material 4 for a more detailed comparison). A 240-second Savitzky–Golay filter was used to correct for scanner drift (Çukur et al. 2013). Furthermore, low-frequency noise and artifacts were attenuated using a Butterworth high-pass filter with cutoff frequency of 0.01 Hz in Nilearn. The filters were applied both to the fMRI data and the fMRIprep confound regressors following recommendations (Lindquist et al. 2019). The Nilearn clean_img function was used to remove the effects of the confounds described below from the BOLD time series to reduce motion and physiological artifacts. The regressors were obtained using fMRIPrep and consisted of 24 head-movement parameters (including their derivatives and powers), eight physiological time series from the WM and CSF (including their derivatives and powers), and motion outliers (Triana et al. 2024). Unlike stated in pre-registration, we did not regress-out confounding effects of heart rate and respiration rate. Finally, Gaussian spatial smoothing with a 6 mm full width at half maximum was applied to the fMRI data using FMRIB Software Library (FSL) (Smith et al. 2004; Woolrich et al. 2009; Jenkinson et al. 2012).

### Regions of interest

We limited our analyses to three pre-registered regions of interest (ROIs) (see pre-registration: osf.io/bfj3u), which have been shown to play a key role in affective and cognitive empathy (Decety and Lamm 2006; Singer and Lamm 2009; Bzdok et al. 2012; Schurz et al. 2021): the aINS, dlPFC, and PCC. More specifically, whereas aINS is key to affective empathy and affective-cognitive integration (Lamm et al. 2011), dlPFC is more related to the top-down affective regulation and perspective-taking of cognitive empathy (Singer and Lamm 2009; Enzi et al. 2016; De Waal and Preston 2017; Fallon et al. 2020), and PCC in contextualizing another perspective with personal experiences (Vogt 2005; Brewer et al. 2013; Fauchon et al. 2019). Crucially, these regions are associated with both empirical (Fan et al. 2011) and theoretical (Jankowiak-Siuda and Zajkowski 2013) accounts of empathy deficits in trait narcissism. The ROIs were defined according to the Brainnetome Atlas (Fan et al. 2016), comprising 210 cortical and 36 subcortical subregions (see atlas.brainnetome.org). The aINS includes four sub-regions of the atlas including left dorsal (BN 167) and ventral (BN 165) agranular, and right dorsal (BN 168) and ventral (BN 166) (see e.g. McDermott et al. 2021). The dlPFC consists of left dorsal area 9/46 (BN 15), ventral area 9/46 (BN 21) and area 46 (BN 19), and right dorsal area 9/46 (BN 16), ventral area 9/46 (BN 22) and area 46 (BN 20) (see e.g. McDermott et al. 2021). The PCC comprises dorsal (BN 175/176), ventral (BN 181/182), and caudal area 23 (BN 185/186) in the left and right hemispheres, respectively (see e.g. Poplin et al. 2023).

### Intersubject correlation analyses

ISC analyses were performed on the BOLD time series recorded while participants viewed the two videos in which another person received negative feedback on their story. These time series were concatenated for ISC computation (total duration of 4 min 15 s). As an additional analysis, the same procedure was applied to two positive feedback videos (total duration of 4 min 7 s; see Supplementary Material 6). ROI-wise average BOLD time series were extracted using the Brainnetome atlas with Nilearn’s NiftiLabelsMasker and z-scored for each participant. Participants were grouped into N+ and N–. For each ROI, pairwise correlations between time series were computed to obtain within- and across-group ISC matrices. Group differences in average ISC were assessed using a permutation-based two-sample *t-*test with the group labels permuted 100,000 times (see Chen et al. 2016). *P*-values were estimated by comparing the observed t-statistics with the null distribution. To control the rate of false positives (Type I errors) across multiple regional comparisons, we applied the Benjamini–Hochberg False Discovery Rate correction. The analysis process is described in Fig. 2.

**Figure 2:**
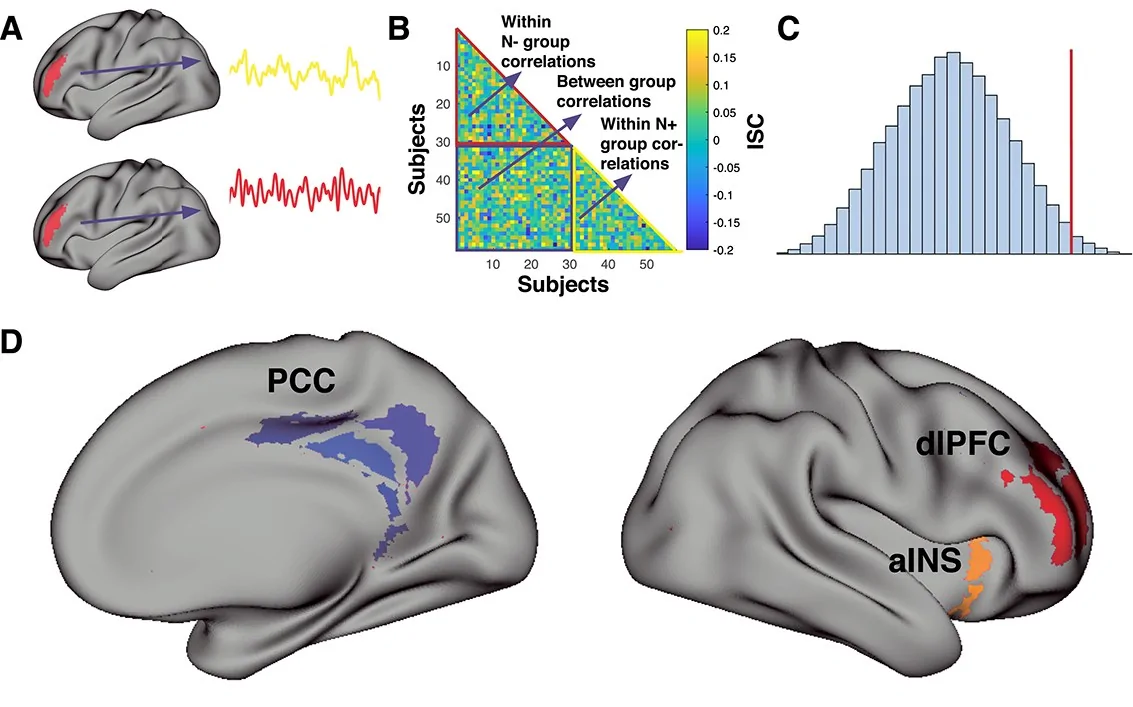
The ISC analysis process. (A) For each region of interest (ROI) correlation between every subject pair’s BOLD timeseries is calculated during viewing of videos in which another person receives face-threatening (negative) feedback. (B) ISC matrix includes correlations between all subject pairs for one ROI. The upper triangle indicates correlations within the low narcissism group, whereas the lower triangle indicates correlations within the high narcissism group. (C) A two-sample *t-*test of difference between groups’ average ISC is calculated, which is compared to permuted distribution. (D) The steps A-C are repeated for every ROI to produce a map of ROIs that show significant differences between the groups. Cortical surface plots were generated using surfplot (Gale et al. 2021) and BrainSpace (Vos de Wael et al. 2020).

### GLM analysis

A GLM analysis was included in this study as a more traditional model-based analysis approach compared to the model-free ISC method. Group differences in BOLD signal strengths between the N+ and N– group in the ROI subregions were compared using GLM. For each subject, a design matrix was constructed in which the regressors of interest corresponded to the time periods when the evaluator’s face-threatening feedback to the other person’s story was most clearly spelled out. We selected these moments because we hypothesized that they would elicit the strongest group differences, as they are most likely to evoke empathic responses in observers. In these moments, the evaluator summarized her view of the story, considering the story as inadequate and/or non-credible. There were three such moments in both face-threatening feedbacks, making together six 15 s segments. The four narratives and admiration feedback videos were not modeled, since the present study focused on negative feedback videos. Nuisance regressors included time points corresponding to the presentation of the SAM questionnaires, instruction periods, waiting periods between tasks and resting state period. Unmodeled segments served as an implicit baseline. The final matrix was convoluted with Hemodynamic Response Function to account for the hemodynamic delay. The matrix was also detrended and confounds were regressed to account for the effect of preprocessing.

### Behavioral and questionnaire analyses

The two-group mean difference comparison of bIRI scores was carried out using the esci package in R (Calin-Jageman 2025). A three-way ANOVA was conducted in R using package rstatix (Kassambara 2023) to examine the effects of group status (two levels), feedback (two levels), and narrative type (two levels) on SAM scores. Self-reported valence, arousal, control and pride during “Other” condition were tested using full factorial three-way ANOVA with Narrative, Feedback (within-subjects) and Narcissism (N+/N–) level of the participant (between-subjects) as independent variables. Multiple comparison adjustment for the post-hoc comparisons was performed using the Bonferroni method.

## Results

### Psychological and behavioral data

Considering the differences between groups in bIRI subscales, the N+ group reported lower empathic concern (bIRI-EC) *(d* = 0.76 95% CI [0.21, 1.29], *P* = .006), personal distress (bIRI-PD) (*d* = 0.76 95% CI [0.22, 1.28], *P* = .005), and perspective taking (bIRI-PT) (*d* = 0.76 95% CI [0.21, 1.28], *P* = .005) compared to the N– group (see Table 1). No significant group difference was found for bIRI-F (*d* = 0.14 95% CI [−0.38, 0.65], *P* = .604).

**Table 1:** Descriptives for the participants included in the analyses.

Group	*N*	Gender F N/%	Age	NPI-13				bIRI			
				Total	GE	EE	LA	PD	PT	F	EC
N+	28	19/67.9%	29.0 (9.08)	46.7 (3.92)	18.1 (2.75)	14.4 (2.13)	14.2 (2.63)	5.32 (3.81)	11.4 (4.43)	12.1 (5.24)	13.0 (4.06)
N–	30	21/70.0%	27.7 (7.16)	21.7 (3.54)	8.47 (2.34)	7.43 (1.50)	5.80 (1.61)	8.23 (3.82)	14.4 (3.47)	12.8 (4.38)	15.5 (2.37)
Total	58	40/70.2%	28.3 (8.10)	33.8 (13.1)	13.1 (5.46)	10.8 (3.97)	9.86 (4.75)	6.83 (4.06)	12.9 (4.21)	12.4 (4.78)	14.3 (3.51)

F N/% = number and percentage of female participants; N+ = High trait Narcissism; N– = Low trait Narcissism; NPI-13 = Narcissistic Personality Index 13, with subscales GE = Grandiose-Exhibitionism; EE = Exploitativeness-Entitlement; LA = Leadership-Authority; bIRI = brief Interpersonal Reactivity Index, with subscales PD = Personal Distress; PT = Perspective Taking; F = Fantasy; EC = Empathic Concern.

### Post feedback ratings

For valence, main effects of Narrative (Admired: *M(SD)* = 6.07(1.78), Excluded: *M(SD)* = 5.22(1.61), F(1,71) = 44.17, *P* < .001), and Feedback (Affirming: *M(SD)* = 6.26(1.66), Threatening: *M(SD)* = 5.03(1.62), F(1,71) = 46.56, *P* < .001) were significant. Narrative × Feedback (F(1,71) = 4.056, *P* = .048) and Narrative × Feedback × Narcissism (F(1,71) = 5.406, *P* = .023) interactions were significant. Affirmative feedback increased valence during admiration narratives, but only for participants with low narcissism levels (N–, F(1,36) = 7.92, *P* = .048, Bonferroni corrected). The main effect of feedback was significant for arousal (F(1,71) = 10.75, *P* = .002) and pride (F(1,71) = 7.82, *P* = .007). Arousal was higher during negative feedback (*M(SD)* = 4.97(1.68)) than during positive feedback (*M(SD)* = 4.40(1.66)), whereas pride was higher during positive feedback (*M(SD)* = 5.75(1.38)) than during negative feedback (*M(SD)* = 5.43(1.25)). See Supplementary Material 5 for additional figures.

### Brain imaging

Group differences in ISCs were observed in the right dorsal area 9/46 (BN 16), the left area 46 (BN 19) and ventral area 9/46 (BN 21) of the dlPFC, and the left and right caudal area 23 (BN 185, 186) of the PCC (see also Supplementary Material 6 Table 1). In these subregions N+ participants showed reduced ISC compared to N– participants. However, only the results for the left ventral area 9/46 (BN 21) of the dlPFC and the right caudal area 23 (BN 186) of the PCC survived when the multiple comparison correction was applied (Fig. 3). Average ISC values in the aINS or its subregions did not differ between the N+ and N– groups when witnessing another person receiving negative feedback. The results of the additional ISC analysis including the positive feedback videos are reported in the Supplementary Material 6 Table 2, with no significant differences observed between groups. Moreover, no group differences were observed in the activation of the ROIs using the GLM analysis.

**Figure 3:**
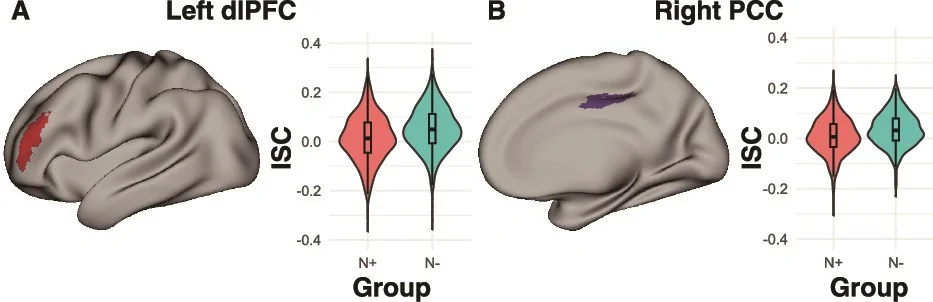
The areas with significantly lower ISC in high narcissistic (N+) compared with low narcissistic (N–) individuals during viewing of videos in which another person received face-threatening (negative) feedback. (A) Left dlPFC (Brainnetome ROI 21). (B) Right PCC (Brainnetome ROI 186). Cortical surface plots were generated using surfplot (Gale et al. 2021) and BrainSpace (Vos de Wael et al. 2020).

## Discussion

This study investigated the neural responses of highly narcissistic and non-narcissistic individuals observing another person receiving face-threatening feedback on personal narratives. Our experimental setting was grounded in Goffman’s (1955, 1956, 1959) conceptualization of the ways in which the self-images of persons are under threat in social interaction and how a face threat can be a source of embarrassment also in the person who is witnessing such threat. We assumed that altered responses to face threats concerning the other might be part of the self-regulatory processing system pertaining to narcissism (Morf and Rhodewalt 2001). Our findings provide novel evidence that narcissism may also be associated with how individuals process others’ experiences of face threat. N+ individuals were neurally less aligned during moments of other-directed face threat. They exhibited lower ISC in the dlPFC and PCC compared to N– individuals. Given the functional roles of these brain regions and our behavioral findings showing that narcissistic traits are associated with reduced self-reported empathy, this weaker neural synchrony observed in N+ individuals may reflect more variable empathic responses to other’s social distress in individuals with high narcissism. In contrast, the N– individuals engaged empathy-related brain areas more coherently. This result supports the idea that narcissistic individuals differ from non-narcissistic persons in the ways they empathize with others’ vulnerable experiences (Urbonaviciute and Hepper 2020; Simard et al. 2023).

More broadly, the present paradigm can be situated within a continuum between third-person and second-person social cognition tasks (Schilbach et al. 2013; Redcay and Schilbach 2019). Unlike paradigms based on static stimuli or naturalistic movie excerpts (Hasson et al. 2010; Jääskeläinen et al. 2021), our task involved an unfolding interpersonal situation in which social meaning emerged dynamically between individuals. From this perspective, neural responses may reflect not only empathy-related processes but also the extent to which participants engaged with a shared interpersonal frame and attuned to the relational dynamics of the interaction. Overall, our findings contribute to a more nuanced understanding of how grandiose narcissism is associated with social cognition in dynamic and socially evaluative contexts.

In line with our preregistered hypothesis, our main finding indicated that the N+ participants show reduced ISC in the left ventral area 9/46 of the dlPFC and the right caudal area 23 of the PCC. The dlPFC and PCC have been consistently implicated in cognitive aspects of empathy (Decety and Lamm 2006; Singer and Lamm 2009; Bzdok et al. 2012; Schurz et al. 2021). Our results align with Fan et al. (2011), who reported reduced right dlPFC and right PCC activation in individuals with high narcissistic traits, encompassing both grandiose and vulnerable forms, during an empathy task focusing on affective resonance when observing pictures of emotional faces. Moreover, our findings are consistent with the self-report data, as N+ individuals reported significantly lower perspective-taking. In contrast to our ISC findings, Jauk et al. (2024), using a naturalistic video-viewing paradigm and a GLM approach, found no significant association between reduced PCC activation and grandiose narcissism, which was also the case in our GLM analyses.

In addition to the dlPFC and PCC, the aINS is a core region involved in empathy, associated primarily with its affective component (Decety and Lamm 2006; Singer and Lamm 2009; Bzdok et al. 2012; Schurz et al. 2021). The aINS responds to the observation of others’ physical pain or emotional distress (Lamm et al. 2011). Although affective empathy often occurs automatically in synchrony with others, it may be compromised in narcissistic individuals due heightened self-focus and -protection (Morf and Rhodewalt 2001; Grijalva and Zhang 2015; Mahadevan et al. 2019; Chen et al. 2024; Mahadevan 2024). While the aINS is activated during self-experienced social stress in individuals with narcissists traits (Cascio et al. 2015), its activity is reduced during empathy tasks (Fan et al. 2011; Scalabrini et al. 2017). Furthermore, Jauk et al. (2024) did not find a significant association between grandiose narcissism and reduced aINS activation at the objective task-contrast level, with effects approaching the corrected significance threshold at the subjective level. Given these findings, the absence of group differences in the aINS in our ISC and GLM analyses was unexpected. It is noteworthy, however, that ISC in the left dorsal agranular insula approached significance (*P* = .052) before correction for multiple comparisons. Our self-report data further showed lower empathic concern in N+ individuals than in the N– group, suggesting a potential group difference in affective empathy. One explanation is that participants may experience well-defined stimuli, such as emotional facial expressions, differently from more complex situations involving people telling stories or receiving feedback, as was the case in both our study and that of Jauk et al. (2024). It is also worth noting that Jauk et al. (2024) examined empathy-inducing narratives involving loss or threat, whereas the present paradigm involved witnessing another person receive face-threatening and potentially embarrassing social evaluation. Furthermore, the present paradigm required participants to observe and interpret a relational social event, in which social meaning emerged through the interaction between individuals, rather than merely respond to a discrete emotional cue or another person’s distress. It is therefore possible that narcissism-related differences were observed in brain areas supporting the interpretation and evaluation of the interaction and not in the aINS more closely associated with affective empathic resonance. Furthermore, Fan et al. (2011) explicitly instructed participants to empathize, and Jauk et al. (2024) included an instruction to relate to the thoughts and feelings of the displayed person, whereas our study did not include such instructions. Encouraging participants to empathize has been shown to influence neural processing of others’ emotions (Borja Jimenez et al. 2020), although such instructions do not appear to fully account for differences across studies in aINS-related findings. Taken together, while empathy-related processes likely contributed to participants’ responses, the narcissism-related differences may also involve variation in how participants engaged with and interpreted the unfolding interpersonal interaction, including the extent to which they attuned to the shared interpersonal frame of the situation.

The realistic nature of the experimental setting offers a novel perspective to narcissism research. These findings underscore the importance of examining personality-related differences in social functioning within more dynamic and naturalistic settings, where social cognitive processes unfold in a manner closer to everyday life. By capturing how the N+ individuals respond to others’ social evaluation, this study contributes to a more nuanced view of empathy. Our study helps to understand how individuals may interpret the same situation in different ways that may potentially lead to misunderstandings and interpersonal difficulties. Ultimately, these insights can inform clinical approaches and support the development of more refined theoretical models of narcissism.

### Strengths, limitations, and need for further research

A novel naturalistic fMRI paradigm advances narcissism research by employing ecological stimuli comprising personal narratives and evaluative feedback to more accurately reflect the complexity of real-world empathic processing and aspects of social interaction specifically associated with narcissism. By introducing ISC analysis, this study applies a method that captures shared neural dynamics during complex social processing, making it particularly well suited for examining real-life social cognition.

Despite these contributions, the study has limitations: 15 participants were excluded due to motion artifacts, and the sample was not fully representative, being largely young, female, and university-recruited. An additional limitation of the present study is that the extreme-groups design did not allow the effects of grandiose and vulnerable narcissism to be disentangled. Consequently, although the findings are associated with grandiose narcissism, we cannot exclude the possibility that elevated vulnerable narcissism also contributed to the observed neural responses. Moreover, because the participants were selected based on grandiose narcissistic traits, the findings cannot be generalized to vulnerable narcissism, which has been more strongly linked to shame, hypersensitivity to evaluation, and social threat concerns. Future studies specifically designed to examine both grandiose and vulnerable narcissism will be better positioned to determine whether the neural responses observed here are specific to grandiosity or extend across narcissistic phenotypes. Some effects were significant before multiple-comparisons correction but did not always survive stricter correction; larger samples in future research would provide greater statistical power. Moreover, while ISC reflects shared neural responses, reduced ISC may reflect multiple processes (e.g. attentional variability, interpretation differences, or disengagement) and not empathy alone. Finally, comparisons across narcissism studies are complicated by heterogeneous measurement tools, highlighting the need for greater standardization and within-study comparisons of instruments in future research.

In addition, as this study used a rather complex design that was quite demanding for the participants, we mostly restricted the self-ratings to well-established methods. However, future studies should more specifically target the dynamic experiences related to face, i.e. embarrassment, humiliation, and other social emotions. It is possible, for instance, that the present findings could be mediated by stronger identification of the participant with the evaluator than the recipient of the feedback. Hence, developing additional behavioral measures and coupling those with the proposed second-person approach offers considerable new opportunities for future research on the topic.

## Conclusion

This study advances research on narcissism and empathy by introducing a naturalistic experimental paradigm emulating real-world situations evoking empathetic behavior. We explored the neural dynamics of empathy in individuals with grandiose narcissistic traits as they observed another person receiving negative feedback on a personal story. Individuals with narcissistic traits were less aligned in their neural responses in the regions associated with cognitive empathy, including the dlPFC and PCC, suggesting greater variability in processing others’ socially threatening experiences. This could reflect the neural mechanism explaining why narcissistic individuals engage less in cognitive empathy. These findings align with prior research and extend it by being the first to demonstrate reduced empathy responses in a complex naturalistic setting using ISC analysis.

Importantly, the use of a naturalistic paradigm highlights the value of studying narcissism and empathy in contexts that more closely resemble real-world interactions, where empathic processes unfold dynamically and are shaped by situational complexity. Future research should extend this approach to other forms of social interaction to better understand how personality-related differences shape the neural processes underlying social perception and interpersonal behavior.

## Supplementary Material

Supplementary_Materials_bhag175

## Data Availability

The data included in this study are available from the corresponding author upon reasonable request.
