# Supplementary material for: Reduced social brain coupling to others’ face threats in narcissism
Source: Cereb Cortex. 2026 Oct 27;36(10):bhag175. doi: 10.1093/cercor/bhag175 (PMC13709960; doi:10.1093/cercor/bhag175)
Supplement: Supplementary file 1 [file supplementary_materials_bhag175.pdf]

Supplementary materials

Reduced social brain coupling to others' face threats in narcissism

Piispanen, M.[1,2,3,4], Tuominen, J.[1,2,5*], Wuolio, M.[2*], Henttonen, P.[6,7], Harjunen, V.J.[8], Koskinen, E.[2,7,8], Krusemark, E.[9], Glerean, E.[1], Rinne, P.[1], Sams, M.[1,4], Peräkylä, A.[2] & Salmi, J.[1,10]

Affiliations

[1]Department of Neuroscience and Biomedical Engineering, Aalto University, Espoo, Finland
[2]Faculty of Social Sciences, University of Helsinki, Helsinki, Finland
[3]Aalto NeuroImaging, Aalto University, Espoo, Finland
[4]MAGICS–Aalto, Aalto University, Espoo, Finland
[5]Department of Psychology and Speech-Language Pathology, University of Turku, Turku, Finland
[6]Helsinki Institute for Social Sciences and Humanities, University of Helsinki, Helsinki, Finland
[7]Faculty of Social Sciences, Tampere University, Tampere; Finland
[8]Faculty of Medicine, University of Helsinki, Helsinki, Finland
[9]Millsaps College, Jackson, Mississippi, United States of America
[10]Unit of Psychology, Faculty of Education and Psychology, University of Oulu, Oulu, Finland
[*]These authors contributed equally to this work.

Corresponding author

Matias Piispanen

Department of Neuroscience and Biomedical Engineering

Aalto University School of Science

P.O. Box 12200

FI-00076 AALTO, Finland

Email: matias.p.piispanen@aalto.fi

Supplementary material 1: Preparation of stimulus materials

We produced videos of simulated “expert feedback” designed either to validate or to challenge the participants' narratives in which they described experiences of being the target of others’ admiration or social exclusion, selected to capture both positively and negatively valenced personally meaningful social experiences. In these feedback videos, an actor portrayed an anthropologist. The feedback was formulated to be naturalistic and sufficiently general to align with a wide range of personal narratives.

As narratives in both admired and excluded conditions received both face-affirming and face-threatening feedback, there were four different feedback videos:

1) admired; face-affirming;

2) admired; face-threatening;

3) excluded; face-affirming;

4) excluded; face-threatening.

In feedback video 1), the admiration described by the narrator was constructed as authentic, credible, and justified. The “expert” presents the narrator as having elicited respect through actions or personal qualities, and the implied audience response is one of endorsement, shared admiration, and positive regard. In feedback video 2), the admiration described in the narrative was constructed as doubtful, misinterpreted, or insufficiently warranted. The “expert” suggests that the apparent admiration may stem from politeness, convention, or projection rather than genuine esteem, and the implied audience response is scepticism rather than identification. In feedback video 3), the narrator’s experience of exclusion is constructed as genuine, credible, and morally warranted. The “expert” frames the narrative as describing unfair, insensitive, or harmful treatment, and the implied audience response is sympathy with the narrator together with recognition that repair or redress would be appropriate. In feedback video 4), the narrator’s experience of exclusion is constructed as exaggerated, weakly grounded, or lacking authenticity. The “expert” suggests that the feeling of exclusion may be self-generated, minor,

or unsupported by the situation, and the implied audience response is reduced sympathy, critical distance, and doubt towards the narrator's own interpretation. First, the feedback scripts were written by a professional scriptwriter and subsequently refined through iterative revisions. Second, a theatre professional was recruited to perform and record the feedback. Based on the recording, additional refinements were implemented, resulting in improved versions.

Next, an amateur actor was recruited to produce four fictional narratives describing experiences of admiration (during adolescence and in the present) and social exclusion (during adolescence and in the present), following the same instructions that were given to the experimental participants. The inclusion of both adolescence and the present narratives ensured that each narrative category could be paired with both positive and negative feedback. The narratives were further revised to enhance their naturalness and relatability for experimental participants.

The actor was then instructed to present these narratives directly to the camera in the same laboratory setting used for participant data collection. Subsequently, the actor was recorded while listening to the expert feedback. She was instructed to respond naturally, without enacting or exaggerating emotional expressions. Finally, the experimental stimulus videos were created through video editing by combining segments of the actor listening to the feedback with the corresponding expert feedback recordings, creating the impression that another participant is receiving the feedback.

Supplementary material 2: Transcriptions of feedback

Positive feedback on the story of being admired by others

Hello, I'm Pärttyli Rinne, an anthropologist, and my task is now to give you feedback on your story.

Hei, mä olen Pärttyli Rinne, antropologi, ja mun tehtäväni on antaa nyt palautetta sulle sun tarinastasi.

In my opinion, it is entirely justified that you have received the kind of admiration you describe.
On mielestäni täysin perusteltua, että sä oot saanut sellaista ihailua, jota sä kuvailet.

Your story gives the impression that the reason others admire you is above all the way your personality manifests in social interactions.
Sun tarinastasi syntyy vaikutelma, että se syy, miksi toiset ihailevat sua, on ennen kaikkea se tapa, millä persoona ilmenee vuorovaikutustilanteissa.

Most likely, your way of encountering others, being present with them, and communicating naturally generates admiration and respect in others.
Todennäköisesti sun tapa kohdata toiset, olla heidän kanssaan läsnä, kommunikoida, luontaisesti synnyttää toisissa ihailua ja kunnioitusta.

In your story, I think there comes through a kind of reflective process in which what is positive and respect-evoking in you is channeled outward.
Sun tarinassa tulee mun mielestä läpi tämmöinen reflektiivinen tapahtuma, jossa se, mikä sussa on myönteistä ja kunnioitusta herättävää, kanavoituu ulos.

And, in a way, it is reflected back from others to you as positivity.
Ja ikään kuin heijastuu toisista positiivisuutena takaisin suhun itseesi.

So, behind the admiration you receive from others is who you are and how you have developed as a person.
Eli sen sun toisilta saaman ihailun taustalla on se, kuka sinä itse olet ja millaiseksi sä olet rakentunut.

People usually have some reason for reacting the way they do.
Ihmisillä on yleensä joku syy reagoida niin kuin ne reagoivat.

In this case, the admiration ultimately, in my view, stems from your personality.

Tässä tapauksessa se ihailu viime kädessä mun mielestä johtuu sun persoonasta.

So, you can be satisfied.

Eli voit olla tyytyväinen.

You deserved that admiration.

Sä ansaitsit sen ihailun.

Negative feedback on the story of being admired by others

I’ll now give you feedback on your story.

Sä saat multa nyt palautteen sun tarinasta.

You describe how your friends have admired you.

Sä kuvaillet, miten sun ystävät on ihailleet sua.

I’m not entirely sure.

Mä en oo, mä en oo aivan varma.

Sometimes we may overinterpret things that happen to us.

Me saatetaan joskus ylitulkita asioita, mitä tapahtuu meille.

For example, to what extent someone actually admires you, even if it feels like they do.

Vaikka, että missä määrin joku tai jotkut nyt sitten oikeasti ihailee, jos susta tuntuu, että ne ihailee sua.

In your story, it may, in my view, also be a matter of a certain wish or to some extent imagined admiration.

Tässä sun tarinassa voi mun nähdäkseni olla kyse myös tietynlaisesta toiveesta tai jossain määrin kuvitellusta ihailusta.

It may be that you have wanted to feel admired or respected.

Voi olla, että sä oot halunnut kokea olevasi ihailtu tai kunnioitettu.

And for that reason, you have experienced it.

Ja sitten tästä syystä kokenut sitä.

Of course, it is also possible and quite natural that in social interactions people may present themselves as more positive than they actually feel.

On tietenkin myös mahdollista ja ihan luonnollista, että joskus ihmiset vuorovaikutustilanteissa saattaa esittää toisille positiivisempaa kuin miltä heistä oikeasti tuntuu.

That is, they may have shown admiration and positivity even if those feelings were not genuinely present.

Eli he saatto osoittaa ihailua ja myönteisyyttä, vaikka ei olisi sitä tunnetta oikeasti ollut siellä taustalla.

In my view, the core of your story is your own desire to be admired.

Mun nähdäkseni tässä sun tarinassa tää sun oma halu tulla ihailluksi on se ydin.

So, you have partly constructed this feeling of being admired in your mind, and whether it was actually true—I'm not fully convinced based on this.

Eli sä oot osittain rakentanut mielessä tätä ihailluksi tulemisen tunnetta ja oliko se sitten totta, niin mä en tämän perusteella oo ihan vakuuttunut.

Negative feedback on the story of being excluded by others

In your story, you describe how you felt left outside the group.

Sä kuvaat tarinassasi, että miten sä oot kokenut jäänees porukan ulkopuolelle.

I get the impression that the underlying cause of this experience may ultimately lie within you.
Mulle synty vaikutelma, että tän kokemuksen pohjasyy on ehkä viime kädessä eniten sussa itsessäsi.

It may be that your approach to the situation—both when it happened and now in this reflective account—is somewhat too one-sided.
Voi olla, että sun lähestymistapa tähän tilanteeseen silloin, kun se tapahtu ja nyt tässä tarinamuotoisessa reflektiossa on ehkä vähän turhan yksioikoinen.

It seems to me that there may be certain blind spots in your behavior and attitudes that you do not fully see yourself.
Mun mielestä vaikuttaa siltä, että sun omassa käyttäytymisessä ja asenteissa on sellaisia ikään kuin sokeita pisteitä, joita sä et itse kunnollisesti näe kenties.

That is understandable—everyone has them—but if your attitudes and behavior do not sufficiently take others' perspectives into account, they may react to you with distance.
Se on ymmärrettävää, kaikilla on, mutta jos sun asenteet ja käytös ei riittävästi huomioi toisten näkökulmaa, niin he saattaa reagoida suhun vierastaen.

In that case, the reason for the exclusion is not outside you in others, but within you.
Ja silloin se syy siihen ulkopuolisuuteen ei ookaan sun ulkopuolella toisissa, vaan se on sun sisällä.

If you adjusted your perspective to be more considerate of others, it would likely be easier for them to take you into account as well.
Jos sä muokkaisit omaa näkökulmaasi enemmän toisia huomioivaksi, niin toisten olis luultavasti helpompi ottaa sut myös paremmin huomioon.

So, in similar situations, you might still work further on your own attitudes.

Eli voisit vastaavissa tilanteissa kenties vielä työstää omia asenteitasi.

Positive feedback on the story of being excluded by others

Your story creates a fairly clear impression of problematic treatment.

Sun kertomus synnyttää aika selkeän vaikutelman ongelmallisesta kohtelusta.

Of course, we all sometimes feel as if we are outside the group.

Meillä kaikilla on tietenkin joskus tunteita, että on ikään kuin porukan ulkopuolella.

But here, in my view, you were left without the respectful consideration that you clearly deserved.

Mutta tässä sut on mun mielestä jätetty vaille sellaista kunnioittavaa huomioimista, joka sulle ilman muuta olis kuulunut.

Your story conveys the impression that the others have, as it were, pushed you aside or excluded you.

Sun tarinasta välittyy kuva, että nää toiset on ikään kuin sysänneet sut syrjään tai sulkeneet sut ulos.

They have not acknowledged your presence.

He eivät ole ottaneet vastaan sun läsnäoloa.

Nor your humanity in that situation.

Ja ihmisyyttä siinä tilanteessa.

Their focus has been on themselves, and this can sometimes also generate ill will.

Huomio on ollut heissä itsessään ja tällainen voi joskus myös synnyttää pahantahtoisuutta.

You should not have been treated that way.

Sua ei olisi pitänyt kohdella noin.

Their behavior is in no way your fault or caused by you.

Heidän käytöksensä ei myöskään ole millään tavalla sun syy tai aiheudu sinusta.

In my view, the others should clearly have acknowledged your presence better and more fairly.

Näiden toisten olisi mun nähdäkseni pitänyt selkeästi paremmin ja reilummin huomioida sun läsnäolo.

In situations like this, even basic common sense suggests that if one realizes they have treated others unfairly, they can also clearly apologize.

Tällaisissa tilanteissa ihan arkijärjelläkin jos huomaa, että on kohdellut toisia epäreilusti, niin voi myös pyytää ihan selkeästi anteeksi.

And in my opinion, that is what you would have deserved from them.

Ja tän sä oisit mun mielestä heiltä ansainnut.

Simply put, you should have been treated with more appreciation, empathy, and understanding.

Yksinkertaisesti sua olisi pitänyt kohdella arvostavammin, empaattisemmin ja ymmärtävämmin.

Supplementary material 3: SAM scales

SAM scale after each feedback segment

Self-Assessment Manikin (SAM: after feedback), pictorial format, scale 1–9:

Thank you for listening. Next, we ask you to assess your current state using four scales.

Kiitos, että kuuntelit. Seuraavaksi pyydämme sinua arvioimaan tämänhetkistä tilaasi neljän asteikon avulla.

Choose the option that best describes your current state on the scale below.

Valitse vaihtoehto, joka parhaiten kuvaa tämänhetkistä tilaasi alla olevalla asteikolla.

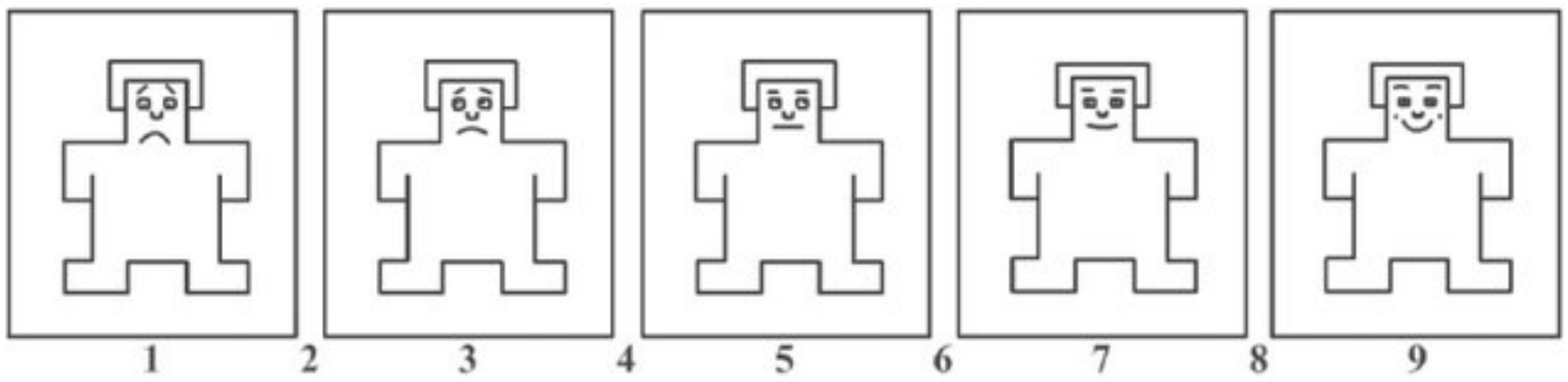

"1: unhappy, dissatisfied, bored --- 9: happy, joyful, satisfied"

"1: onneton, tyytymätön, ikävystynyt --- 9: onnellinen, iloinen, tyytyväinen"

Choose the option that best describes your current state on the scale below.

Valitse vaihtoehto, joka parhaiten kuvaa tämänhetkistä tilaasi alla olevalla asteikolla.

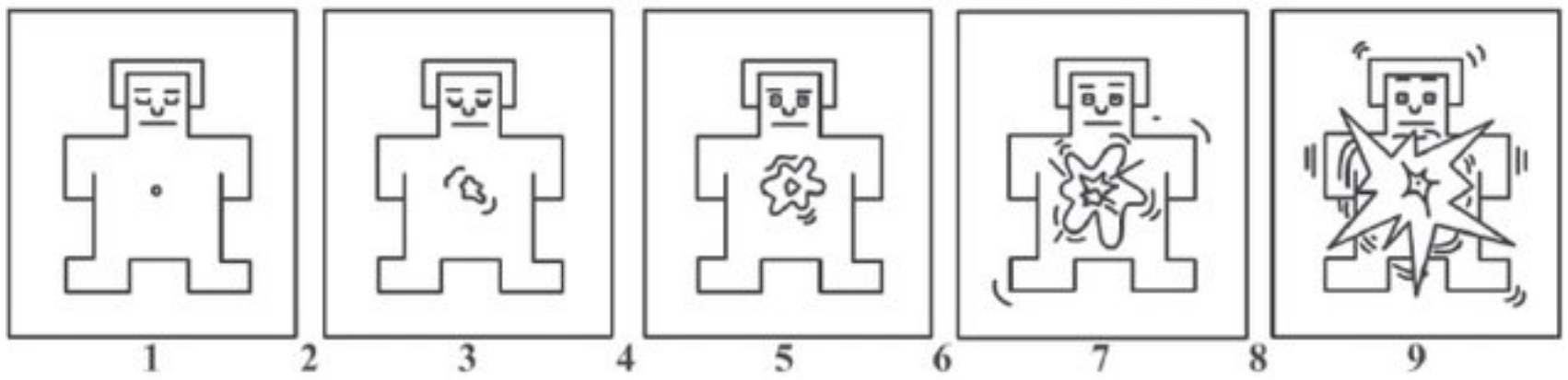

"1: calm, sleepy, sluggish --- 9: energetic, aroused, alert"

"1: tyyni, unelias, raukea --- 9: energinen, kiihtynyt, valpas"

Choose the option that best describes your current state on the scale below.

Valitse vaihtoehto, joka parhaiten kuvaa tämänhetkistä tilaasi alla olevalla asteikolla.

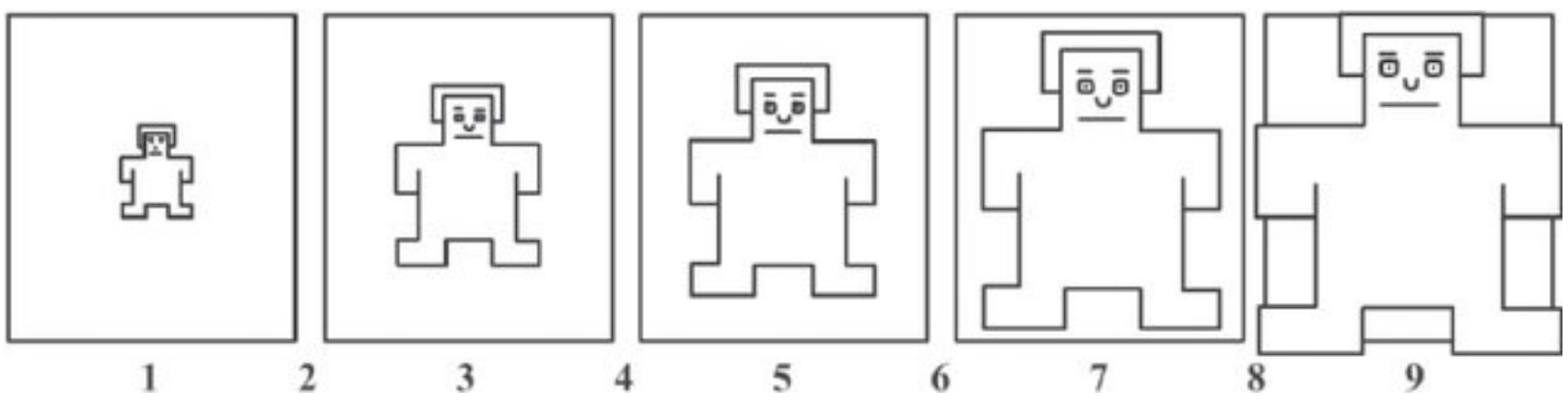

"1: helpless, at the mercy of the situation --- 9: capable, in control of the situation"

"1: avuton, tilanteen armoilla --- 9: pystyvä, tilanne hallinnassa"

Choose the option that best describes your current state on the scale below.

Valitse vaihtoehto, joka parhaiten kuvaa tämänhetkistä tilaasi alla olevalla asteikolla.

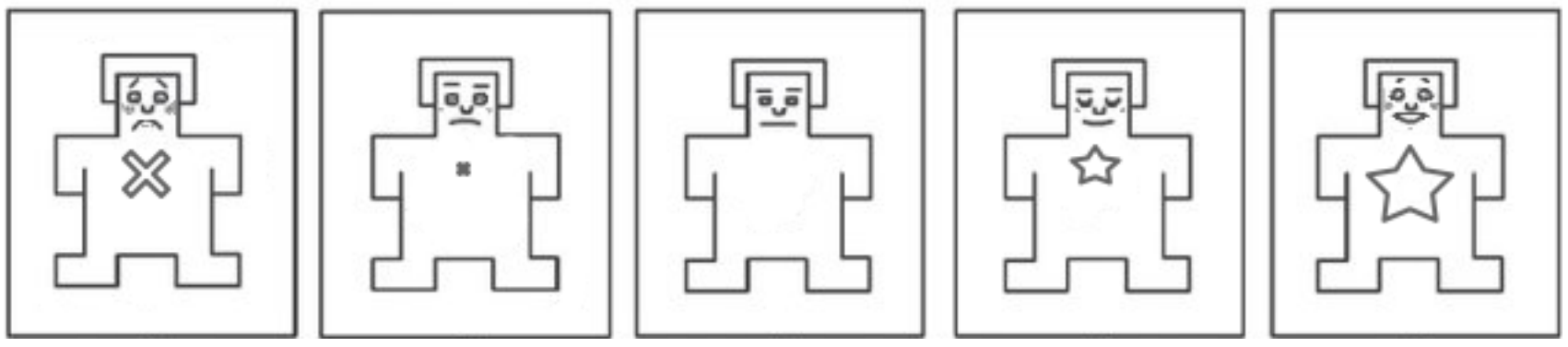

"1: shame --- 9: pride"

"1: häpeä --- 9: ylpeys"

Supplementary material 4: Comparison of the included and excluded groups

To investigate whether the participants excluded based on excessive head motion differed from those included in the analyses we compared these groups. The included/excluded groups did not differ in terms of age (*d* = -0.026, 95 % CI [-0.594, 0.542]) nor narcissism scores (*d* = 0.124, 95 % CI [-0.444, 0.692]). Further examining the subscores in the brief interpersonal reactivity index (bIRI) there were no differences in personal distress (*d* = -0.023, 95 % CI [-0.591, 0.545]), empathic concern (d = -0.318, 95 % CI [-0.888, 0.254]), or fantasy (*d* = -0.504 95 % CI [-1.075, 0.072]), while there was a difference in perspective taking (*d* = -0.775, 95 % CI [-1.354, -0.191]). We therefore investigated this difference further to estimate whether it differentially affected either the N- or N+ category, and while it more strongly affected the N- than the N+ group, this narcissism group x inclusion difference in differences interaction nonetheless was found to be non-significant (*d* =1.075 95 % CI [-0.080, 2.223]).

Supplementary material 5: Rating of clips

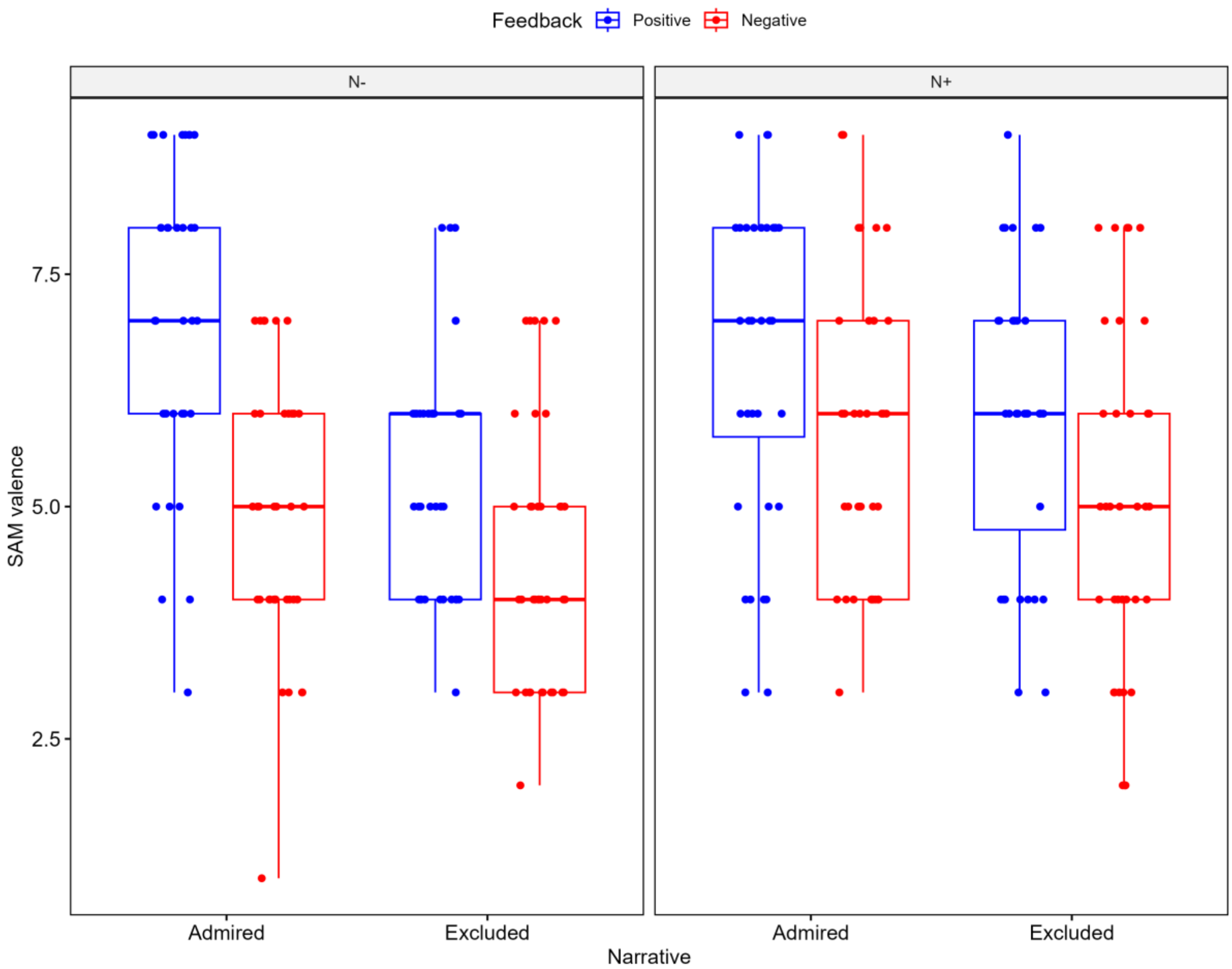

Supplementary Figure 1. Boxplots and individual values of SAM valence between high (N+) and low (N-) narcissistic participants after viewing narratives in which the protagonist was either admired or excluded, and subsequently received positive or negative feedback. Lower values indicate lower valence, whereas higher values indicate higher valence.

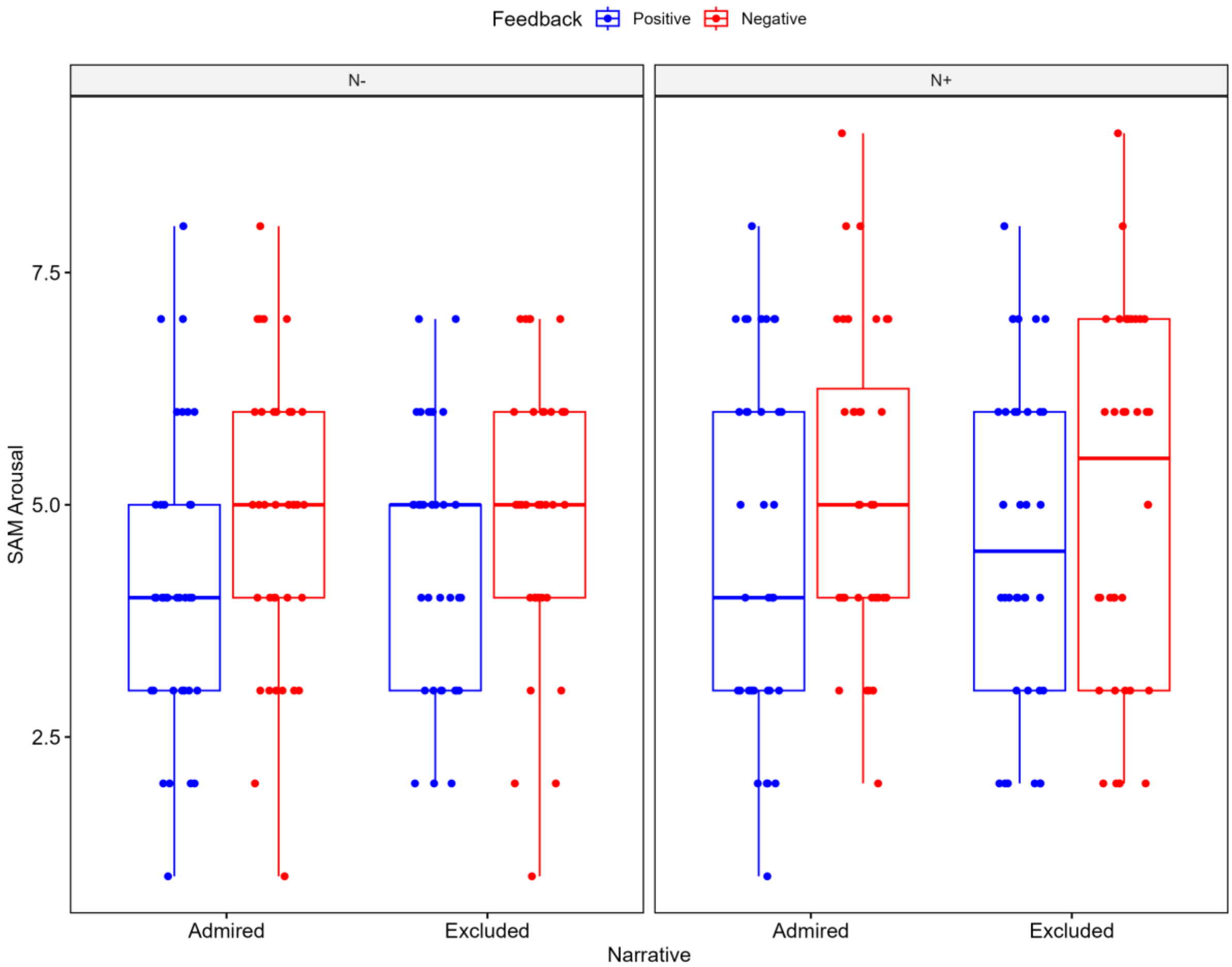

Supplementary Figure 2. Boxplots and individual values of SAM arousal between high (N+) and low (N-) narcissistic participants after viewing narratives in which the protagonist was either admired or excluded, and subsequently received positive or negative feedback. Lower values indicate lower arousal, whereas higher values indicate higher arousal.

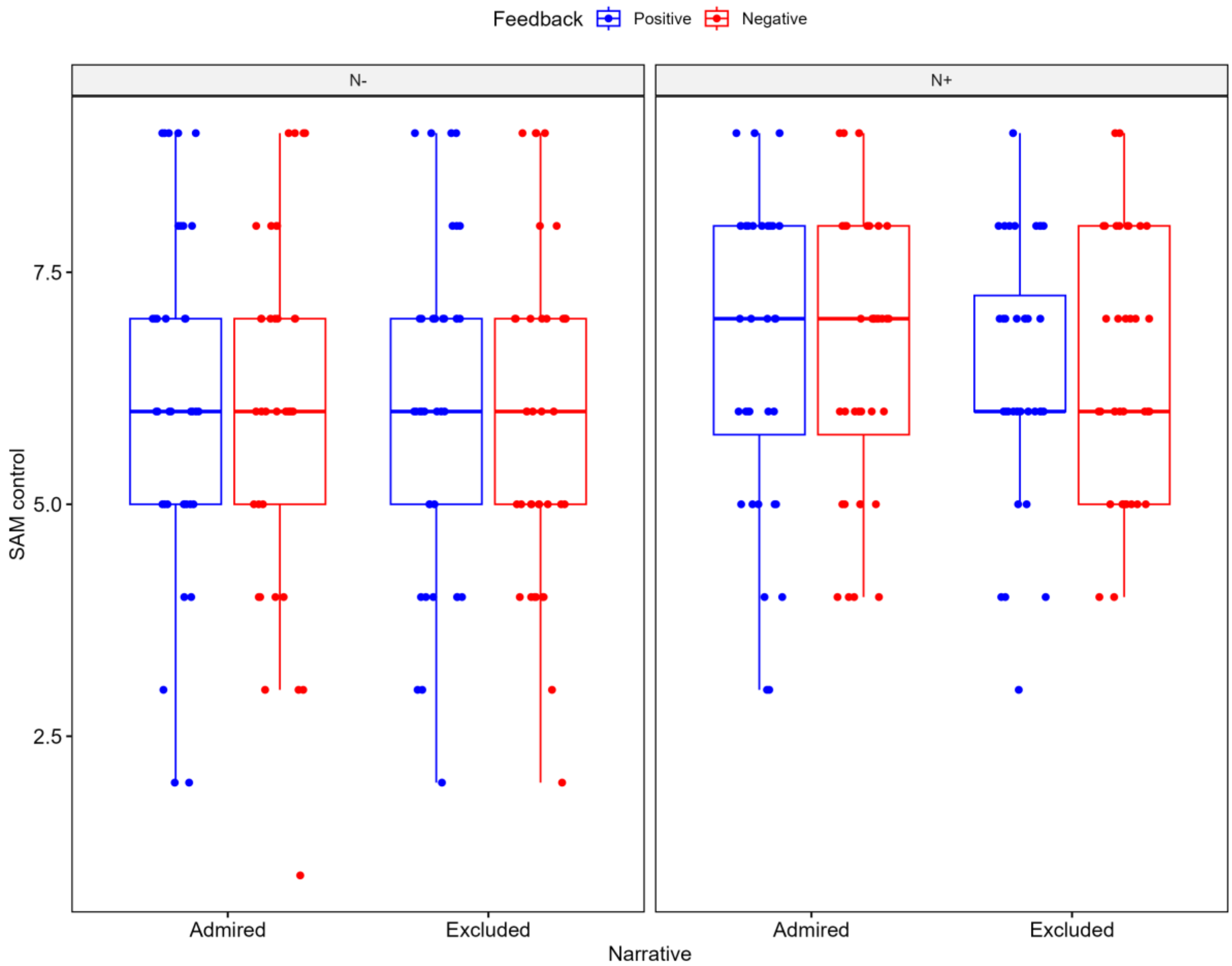

Supplementary Figure 3. Boxplots and individual values of SAM control between high (N+) and low (N-) narcissistic participants after viewing narratives in which the protagonist was either admired or excluded, and subsequently received positive or negative feedback. Lower values indicate greater feelings of helplessness, whereas higher values indicate greater feelings of being capable and in control of the situation.

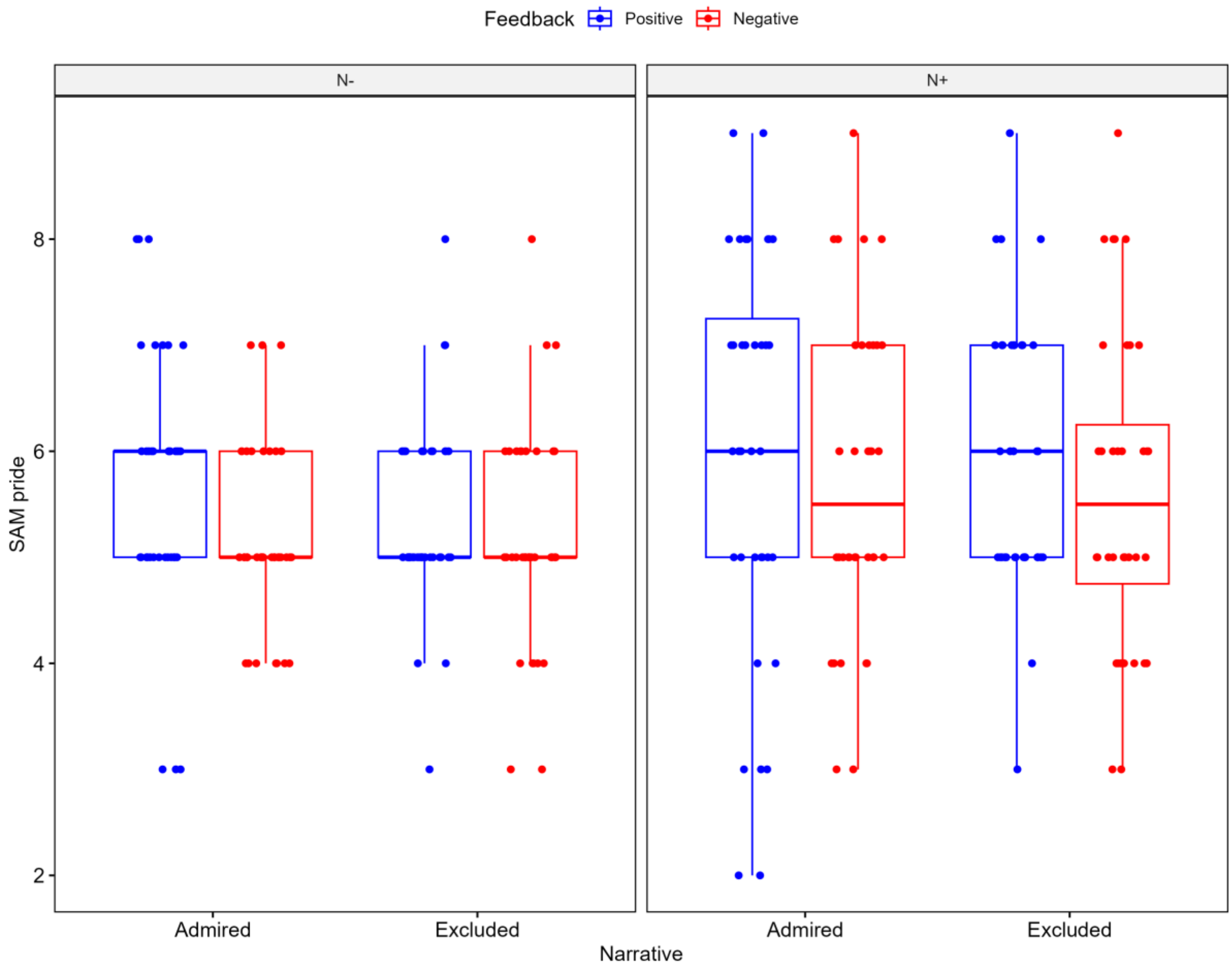

Supplementary Figure 4. Boxplots and individual values of SAM pride between high (N+) and low (N-) narcissistic participants after viewing narratives in which the protagonist was either admired or excluded, and subsequently received positive or negative feedback. Lower values indicate experienced shame and higher values pride.

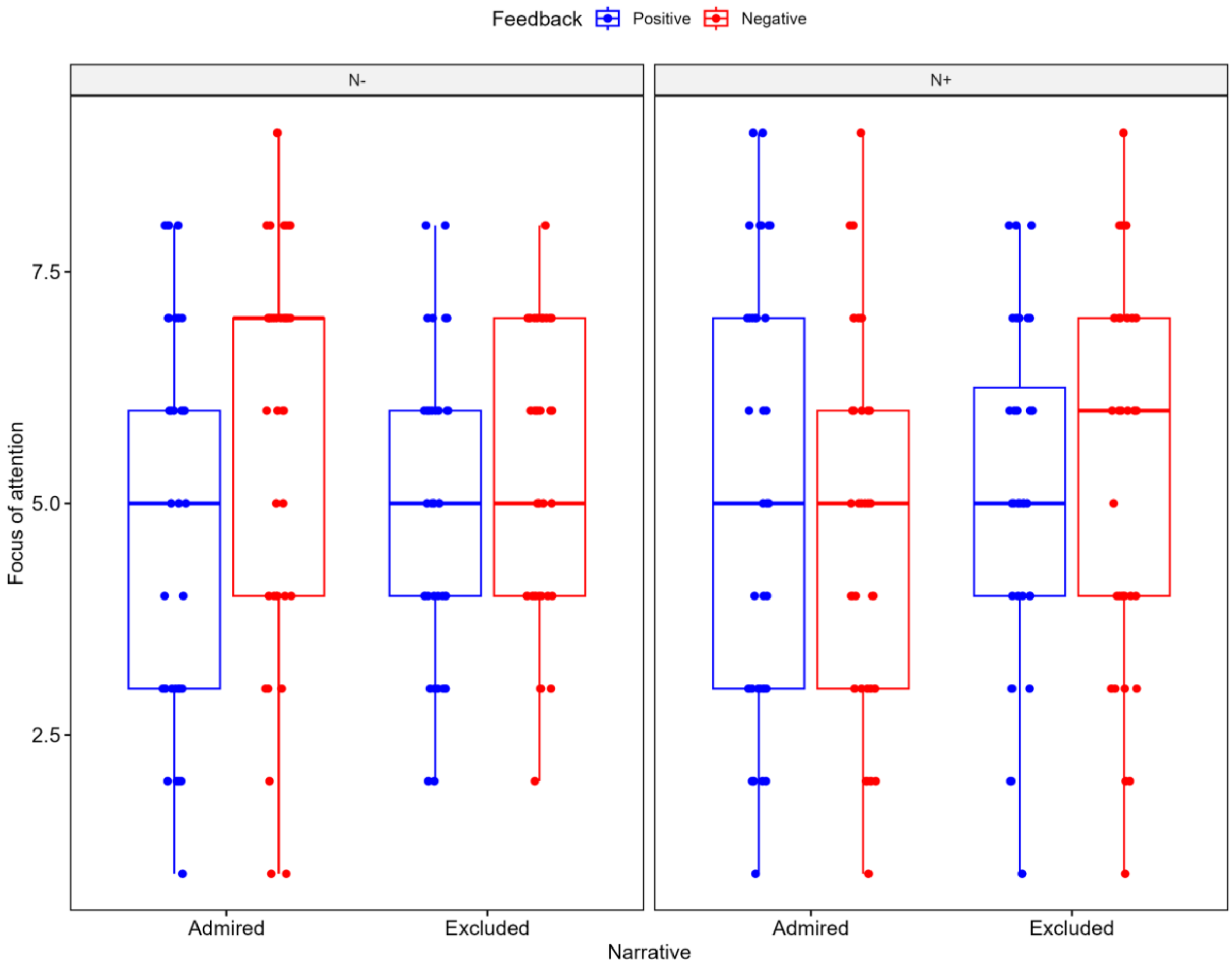

Supplementary Figure 5. Boxplots and individual values of Focus of Attention for high (N+) and low (N−) narcissistic participants after viewing narratives in which the protagonist was either admired or excluded and subsequently received positive or negative feedback. Participants rated the question: "During the feedback, was your attention focused more on the feedback provider or the narrator?" Ratings ranged from 1 = attention focused on the feedback provider (evaluator) to 9 = attention focused on the narrator (story protagonist). Thus, higher values indicate greater attention to the narrator, whereas lower values indicate greater attention to the feedback provider.

Supplementary material 6: Description of the regions of interest and results of the ISC comparison

Supplementary Table 1: Description of the regions of interest and results of the ISC comparison for negative feedbacks

Anatomical location	Brainnetome label ID	MNI Coordinates	T-value	P-value (BHFDR cor.)
Left anterior insula	165	-32, 14, -13	1.6662	0.3011
	167	-34, 18, 1	2.9615	0.1522
Right anterior insula	166	33, 14, -13	0.4634	0.4020
	168	36, 18, 1	0.7992	0.3908
Left dorsolateral prefrontal cortex	15	-27, 43, 31	1.7715	0.3011
	19	-28, 56, 12	2.9808	0.1487
	21	-41, 41, 16	5.8725	0.0356*
Right dorsolateral prefrontal cortex	16	30, 37, 36	5.0703	0.0694
	20	28, 55, 17	1.4041	0.3203
	22	42, 44, 14	3.4540	0.1522
Left posterior cingulate cortex	153	-6, -55, 34	-0.6592	0.3908
	175	-4, -39, 31	-0.5014	0.3908
	181	-8, -47, 10	0.9776	0.3203
	185	-7, -23, 41	3.2275	0.0694
Right posterior cingulate cortex	154	6, -54, 35	-1.1127	0.3908
	176	4, -37, 32	1.6558	0.2770
	182	9, -44, 11	-0.2262	0.4338
	186	6, -20, 40	5.2813	0.0356*

Note. Positive t-values indicate higher ISC in individuals with low narcissistic traits compared to those with high narcissistic traits. BHFDR cor. = Benjamini–Hochberg False Discovery Rate correction. *Significant at p < .05

Supplementary Table 2: Description of the regions of interest and results of the ISC comparison for positive feedbacks

Anatomical location	Brainnetome label ID	MNI Coordinates	T-value	P-value (BHFDR cor.)
Left anterior insula	165	-32, 14, -13	0.0647	0.4952
	167	-34, 18, 1	0.6833	0.4952
Right anterior insula	166	33, 14, -13	-1.1153	0.4952
	168	36, 18, 1	2.4483	0.4952
Left dorsolateral prefrontal cortex	15	-27, 43, 31	-0.8107	0.4952
	19	-28, 56, 12	0.1639	0.4952
	21	-41, 41, 16	2.4251	0.4952

Right dorsolateral prefrontal cortex	16	30, 37, 36	0.5794	0.4952
	20	28, 55, 17	0.0309	0.4952
	22	42, 44, 14	0.9743	0.4952
Left posterior cingulate cortex	153	-6, -55, 34	-1.8587	0.4952
	175	-4, -39, 31	-1.8661	0.4952
	181	-8, -47, 10	1.1942	0.4952
	185	-7, -23, 41	-0.3417	0.4952
Right posterior cingulate cortex	154	6, -54, 35	-1.5060	0.4952
	176	4, -37, 32	-1.9923	0.4952
	182	9, -44, 11	-0.2182	0.4952
	186	6, -20, 40	0.1353	0.4952

Note. Positive t-values indicate higher ISC in individuals with low narcissistic traits compared to those with high narcissistic traits. BHFDR cor. = Benjamini–Hochberg False Discovery Rate correction.
